# Expression Profiles of Branchial FXYD Proteins in the Brackish Medaka *Oryzias dancena*: A Potential Saltwater Fish Model for Studies of Osmoregulation

**DOI:** 10.1371/journal.pone.0055470

**Published:** 2013-01-31

**Authors:** Wen-Kai Yang, Chao-Kai Kang, Chia-Hao Chang, An-Di Hsu, Tsung-Han Lee, Pung-Pung Hwang

**Affiliations:** 1 Department of Life Sciences, National Chung Hsing University, Taichung, Taiwan; 2 Department of Biotechnology, MingDao University, Changhua, Taiwan; 3 Department of Biological Science and Technology, China Medical University, Taichung, Taiwan; 4 Institute of Cellular and Organismic Biology, Academia Sinica, Taipei, Taiwan; University of Tokyo, Japan

## Abstract

FXYD proteins are novel regulators of Na^+^-K^+^-ATPase (NKA). In fish subjected to salinity challenges, NKA activity in osmoregulatory organs (e.g., gills) is a primary driving force for the many ion transport systems that act in concert to maintain a stable internal environment. Although teleostean FXYD proteins have been identified and investigated, previous studies focused on only a limited group of species. The purposes of the present study were to establish the brackish medaka (*Oryzias dancena*) as a potential saltwater fish model for osmoregulatory studies and to investigate the diversity of teleostean FXYD expression profiles by comparing two closely related euryhaline model teleosts, brackish medaka and Japanese medaka (*O. latipes*), upon exposure to salinity changes. Seven members of the FXYD protein family were identified in each medaka species, and the expression of most branchial *fxyd* genes was salinity-dependent. Among the cloned genes, *fxyd11* was expressed specifically in the gills and at a significantly higher level than the other *fxyd* genes. In the brackish medaka, branchial *fxyd11* expression was localized to the NKA-immunoreactive cells in gill epithelia. Furthermore, the FXYD11 protein interacted with the NKA α-subunit and was expressed at a higher level in freshwater-acclimated individuals relative to fish in other salinity groups. The protein sequences and tissue distributions of the FXYD proteins were very similar between the two medaka species, but different expression profiles were observed upon salinity challenge for most branchial *fxyd* genes. Salinity changes produced different effects on the FXYD11 and NKA α-subunit expression patterns in the gills of the brackish medaka. To our knowledge, this report is the first to focus on FXYD expression in the gills of closely related euryhaline teleosts. Given the advantages conferred by the well-developed Japanese medaka system, we propose the brackish medaka as a saltwater fish model for osmoregulatory studies.

## Introduction

Teleosts of the medaka genus *Oryzias* were recently reported to come from different natural habitats (e.g., river, estuary, or ocean) [1], [2] and to have diverse osmoregulatory capabilities [3], [4]. Among *Oryzias* species, the brackish medaka (*Oryzias dancena*) is native to brackish water (BW) environments, such as river mouths and estuaries [1], [2], [5], and exhibits better adult survival and hatching rates of fertilized eggs in hyperosmotic environments (i.e., seawater (SW)) as compared with the Japanese medaka (*O. latipes*) [3], [4]. Along with the zebrafish (*Danio rerio*), Japanese pufferfish (*Takifugu rubripes*), and spotted green pufferfish (*Tetraodon nigroviridis*), the Japanese medaka is an important model teleost with genomic sequences available for molecular studies. However, the zebrafish is a stenohaline species, and the two pufferfish species are not easy to breed in the laboratory. In contrast, the Japanese medaka is a euryhaline freshwater (FW) teleost [6], [7] that is closely related to the brackish medaka [4], [7]. The brackish medaka, like the Japanese medaka, is characterized by a small size, easy breeding, short generation time, high fecundity, and transparent eggs suitable for studies of embryogenesis as well as transgenic experiments [4], [8], [9]. FW fish models such as the zebrafish and Japanese medaka were established and have been widely studied in the post-genomic era, but a suitable euryhaline BW/SW fish model that can be easily bred in the laboratory and has a usable genomic database and standard molecular resources is still lacking. Given that the entire genome of the Japanese medaka has been sequenced and assembled, sufficient biological information can be extrapolated to the brackish medaka [10], [11] to support its use as a model to study the effects of salinity on various *in vivo* molecular expression [12]–[14]. The brackish medaka may therefore represent a good teleost model for osmoregulatory studies in saltwater environments (including BW and SW), but little is known about how their osmoregulatory systems respond to salinity challenges.

The fish Na^+^-K^+^-ATPase (NKA) is a primary driving force for the many ion transport systems activated during a salinity challenge in osmoregulatory organs such as the gills [15], [16]. NKA is a P-type ATPase consisting of an (αβ)_2_ protein complex (the catalytic α-subunit and the smaller glycosylated β-subunit) [17], [18]. In humans, NKA enzymatic activity is generated and maintained by ATP hydrolysis, accounting for approximately 30% of the total energy consumption [19], [20]. In fish, the gill is the major organ responsible for osmoregulation and ionoregulation [15], [21], and most euryhaline teleosts exhibit adaptive alterations in branchial NKA activity following salinity changes [16], [21], [22]. In gill epithelial cells, most of the NKA detected by immunostaining is localized to mitochondrion-rich cells (MR cells; i.e., chloride cells or ionocytes) [15], [16], [21]. Kang et al. [12] reported that the brackish medaka and the Japanese medaka have different salinity effects on branchial NKA profiles in terms of mRNA levels, protein abundance, enzymatic activity, and the size/number of NKA-immunoreactive (IR) cells. These results indicated that the different osmoregulatory capabilities of these two closely related species may be associated with distinct adaptive responses in branchial NKA activity. It should therefore be interesting to compare modulating mechanisms of NKA responses to salinity challenges between the brackish medaka and Japanese medaka. In general, in the cells engaged in transepithelial ion transport, elevated NKA activity provides a greater driving force for ion transport, indicating an increased demand for ion uptake or secretion [21], [23]. NKA expression and/or activity should therefore be modulated by a variety of mechanisms under changing physiological conditions [24].

In the past decade, the FXYD (FXYD domain-containing ion transport regulator) proteins, named by their invariant FXYD motif [25], were found to be novel regulators of NKA [18], [24], [26]. Mammalian members of the FXYD protein family are small ubiquitous membrane proteins with a transmembrane domain and an extracellular FXYD motif that is required for the structural interaction with NKA [18], [19], [25]. The mammalian FXYD protein family consists of at least seven members, including FXYD1 (or phospholemman, PLM), FXYD2 (or the γ-subunit of NKA), FXYD3 (or mammary tumor-associated 8-kDa protein, Mat-8), FXYD4 (or corticosteroid hormone-induced factor, CHIF), FXYD5 (or related to ion channel, RIC; or dysadherin), FXYD6 (or phosphohippolin), and FXYD7 [24], [26], [27]. The mammalian FXYD proteins exhibit tissue-specific distribution, and all mammalian FXYD proteins are known to interact with NKA and affect its kinetic properties in specific ways [26]–[28]. The first reported FXYD protein in fish was a phospholemman-like protein (i.e., FXYD10) from the piked dogfish (*Squalus acanthias*) [29]. Subsequent studies revealed the function of FXYD10 and its interaction with NKA [30], [31].

In teleosts, an interaction between a FXYD and NKA in the gills of the spotted green pufferfish was first reported by Wang et al. [32]. Meanwhile, multiple FXYD proteins (e.g., FXYD2, 5–9, 11, and 12) have been reported in the zebrafish and the Atlantic salmon (*Salmo salar*) [33], [34]. Salinity-dependent mRNA and/or protein expression of branchial FXYD members suggested their physiological significance in osmoregulatory acclimation and a role in modulating the kinetic properties of NKA [32], [34]–[38]. Although the expression and function of FXYD proteins have been widely studied in mammals and sharks, only a few studies have examined the properties of these proteins in a limited group of euryhaline teleosts. No study has yet compared the expression of FXYD proteins in related teleost species acclimated to different salinities.

We identified novel members of the FXYD family in two closely related euryhaline medaka species (brackish medaka and Japanese medaka) that show different branchial NKA responses to salinity challenge. We show that FXYD11 is a gill-specific FXYD protein and that the branchial *fxyd11* mRNA abundance was significantly higher than that of other *fxyd* genes. Our observations indicated the possibility that branchial FXYD proteins exhibited different expression patterns in the two medaka species. The present study investigated the mRNA expression patterns of *fxyd* genes in gills (the major osmoregulatory organ) of medaka acclimated to different salinity environments. By comparing the expression patterns of branchial FXYD proteins between two closely related medaka species with distinct osmoregulatory capabilities, the present study revealed different effects of salinity on teleost FXYD protein profiles. Furthermore, to study the protein levels of gill-specific FXYD11 and its interaction with NKA, we designed an antibody against the FXYD11 protein cloned from brackish medaka. To our knowledge, this report is the first to investigate FXYD expression in the gills of closely related euryhaline teleosts. The results of this study support the use of brackish medaka as a potential saltwater fish model for osmoregulatory studies.

## Materials and Methods

### Ethics Statement

The protocol used for the experimental fish was reviewed and approved by the Institutional Animal Care and Use Committee of the National Chung Hsing University (IACUC approval no. 96-48 to THL).

### Experimental animals and environments

Adult brackish medaka, identified by sequencing the 12S and 16S mitochondrial rRNA genes [39], were obtained from a local aquarium and were 2.6±0.2 cm in standard length. Adult reddish orange Japanese medaka (HI strain), approximately 2.5±0.3 cm standard length, were inbred in the laboratory. Brackish medaka and Japanese medaka were maintained in BW and FW, respectively. For experiments, the two medaka species were acclimated to either FW, BW, or SW for at least four weeks at 28±1°C in a 14-h light: 10-h dark cycle [12]–[14]. BW (15‰) and SW (35‰) were prepared from aerated dechlorinated tap FW by adding standardized amounts of the synthetic sea salt “Instant Ocean” (Aquarium Systems, Mentor, OH, USA). The details of the water parameters were identical to those used in previous studies [13], [40]. The water was continuously circulated through fabric-floss filters and partially refreshed every week. Dead fish were immediately removed from the experimental tanks to maintain the water quality. Fish were fed a daily diet of commercial pellets *ad libitum*. In the following experiments, fish were not fed and were anesthetized with MS-222 (100–200 mg/L) before sampling. Sampled fish were then analyzed as described below.

### Total RNA extraction

The methods used in this study were modified from our previous studies [12]–[14]. Total RNA samples from various tissues and organs of the two medaka species were extracted using RNA-Bee™ (Tel-Test, Friendwood, TX, USA) following the manufacturer's instructions. Genomic DNA was eliminated using the RNA clean-up protocol of the RNAspin Mini RNA isolation kit (GE Health Care, Piscataway, NJ, USA). RNA integrity was verified by 0.8% agarose gel electrophoresis. Extracted RNA samples were stored at −80°C after isolation. The concentration of the extracted RNA was measured with a NanoDrop 2000 (Thermo, Wilmington, DE, USA).

### cDNA cloning of full-length *fxyd* genes

For RACE (rapid amplification of cDNA ends) PCR, extracted RNA samples were prepared as described above. cDNA for cloning and RACE was prepared from total RNA samples isolated from various tissues and organs (i.e., gills, kidneys, intestines, and brains) using the SuperScript III reverse transcriptase kit (Invitrogen, Carlsbad, CA, USA) and the SMART RACE cDNA amplification kit (Clontech, Palo Alto, CA, USA) following the manufacturers' protocols. For PCR amplification, 2 µL of cDNA was used as a template in a 50 µL reaction containing 0.25 mM dNTPs, 2.5 U Hot start EX-Taq polymerase (Takara, Shiga, Japan), and 0.2 µM of each primer. Specific primer sets for RACE PCR are listed in Table S4. PCR products were subcloned into the pOSI-T vector (Genemark, Taipei, Taiwan), and amplicons were sequenced to confirm the PCR products.

### FXYD sequences

To characterize the FXYD sequences, we applied the bioinformatic tools available at the National Center for Biotechnology Information (http://www.ncbi.nlm.nih.gov/) and the ExPASy proteomics server of the Swiss Institute of Bioinformatics (http://www.expasy.org/sprot/). The sequenced *fxyd* genes were deduced for open reading frames by ORF Finder (http://www.ncbi.nlm.nih.gov/gorf/gorf.html). Amino acid sequences for FXYD proteins from different organisms were aligned with BioEdit version 7.0.5.3 [41], and a phylogenetic tree was constructed with MEGA 5 [42] using the minimum evolution method. The SignalP 4.0 server was used to predict the presence and location of signal peptide cleavage sites in the amino acid sequences (http://www.cbs.dtu.dk/services/SignalP/) [43]. Predictions of putative transmembrane segments were performed with the SOSUI server 1.11 (http://bp.nuap.nagoya-u.ac.jp/sosui/) [44]. Potential phosphorylation sites were predicted by the NetPhos 2.0 server (http://www.cbs.dtu.dk/services/NetPhos/) [45]. Potential O- and N-glycosylation sites were predicted by the NetOglyc 3.1 server (http://www.cbs.dtu.dk/services/NetOGlyc/) [46] and NetNGlyc 1.0 server (http://www.cbs.dtu.dk/services/NetNGlyc/) [47], respectively.

### Preparation of tissue cDNA

For reverse transcription (RT), extracted RNA samples were prepared as previously described. The first-strand cDNA was subsequently synthesized by reverse-transcribing 5 µg of total RNA using 1 µL Oligo-dT (0.5 µg/µL) primer and 1 µL PowerScript™ Reverse Transcriptase (Clontech), according to the manufacturer's instructions. The cDNA products were stored at −20°C until analysis by RT-PCR and quantitative real-time PCR (Q-PCR).

### Analysis of *fxyd* genes in different tissues

The expression of *fxyd* genes in various organs of the two medaka species was examined by RT-PCR. Primers were designed to target specific regions of the *fxyd* genes identified in the cloning results (Table S4). For PCR amplification (40 cycles), 1 µL of cDNA was used as a template in a 25 µL final reaction volume containing 0.25 µM dNTPs, 1.25 U Hot start EX-Taq polymerase (Takara), and 0.2 µM of each primer. The bands of PCR products were obtained by 0.8% agarose gel electrophoresis. Moreover, the PCR products were subjected to subcloned into the pOSI-T vector (Genemark) and sequenced for confirmation. Ribosomal protein L7 (RPL7/*rpl7*) was used as an internal control for all tissues (Table S4).

### Quantitative real-time PCR (Q-PCR)

Q-PCR was performed using a MiniOpticon real-time PCR system (Bio-Rad, Hercules, CA, USA). PCR reactions contained 8 µL of cDNA (1000× dilution), 2 µL of either 1 µM *fxyd*-QPCR primers or 1 µM *rpl7* primers (as an internal control), and 10 µL of 2× SYBR Green Supermix (Bio-Rad). The Q-PCR analysis and the formula used to calculate target gene expression were described by Kang et al. [12], [14]. Probe constructs for Q-PCR are shown in Tables S4 and S5. The amplification efficiency of all used primer pairs was similar (ranges in 94.4–103.5% and 94.6–103.9% of the primer pairs in the brackish medaka and Japanese medaka, respectively).

### Antiserum/antibody

The primary antisera/antibodies used in the present study included (1) FXYD11: an affinity-purified rabbit polyclonal antiserum made against a specific epitope (NQCARLVRGKRSDSSSA) corresponding to the C-terminal region of the FXYD11 protein cloned from brackish medaka (LTK BioLaboratories, Taoyuan, Taiwan), used for immunoblotting (1∶5000 dilution) and immunoprecipitation; (2) NKA: a mouse monoclonal antibody (α5, Developmental Studies Hybridoma Bank, Iowa City, IA, USA) raised against the α-subunit of the avian NKA, used for immunoblotting (1∶5000 dilution), immunohistochemical (IHC) staining (1∶400 dilution), and immunoprecipitation; and (3) RPL7: a rabbit polyclonal antibody (ab72550, Abcam, Cambridge, UK) raised against the human RPL, applied as a loading control for immunoblotting (1∶10000 dilution). The secondary antibodies used for immunoblots were horseradish peroxidase (HRP)-conjugated goat anti-mouse IgG or goat anti-rabbit IgG (#0031430 or #0031460, respectively; Pierce, Rockford, IL, USA).

### Preparation of tissue homogenates for immunoblotting and immunoprecipitation

The methods used in this study to prepare tissue homogenates were modified from our previous studies [14], [48]. Various tissues and organs of the two medaka species, including gills, kidneys, and intestines, were dissected and stored immediately in a microcentrifuge tube at −80°C. Sample scrapings were suspended in a mixture of homogenization medium (SEID buffer; 150 mM sucrose, 10 mM EDTA, 50 mM imidazole and 0.1% sodium deoxycholate, pH 7.5) containing proteinase inhibitor (#11836145001, Roche, Indianapolis, IN, USA)(v/v: 25/1). Homogenization was performed in 2 mL microtubes with a Polytron PT1200E (Kinematica, Lucerne, Switzerland) at maximal speed for 30 sec on ice. The homogenate was then centrifuged at 5,000×*g* at 4°C for 5 min. The sample supernatants were collected and used to measure protein concentrations for immunoblotting or immunoprecipitation. Protein concentrations were determined with the BCA Protein Assay (Pierce) using bovine serum albumin (BSA; Pierce) as a standard. Supernatants were stored at −80°C prior to use.

### Immunoblotting

The immunoblotting protocol used herein was modified from our previous studies [32], [40], [48]. For immunoblotting of branchial FXYD11, aliquots containing 40 µg of branchial supernatants were added to sample buffer and heated at 60°C for 15 min followed by electrophoresis on a 15% sodium dodecyl sulfate-polyacrylamide gel. The pre-stained protein molecular weight marker was purchased from Invitrogen (LC5925, Carlsbad, CA, USA). Separated proteins were transferred from unstained gels to PVDF membranes (Millipore, Bedford, MA, USA) by electroblotting using a tank transfer system (Mini Protean 3, Bio-Rad). Blots were preincubated for 2 h in PBST (phosphate buffer saline with 0.05% Tween-20) buffer containing 5% (wt/vol) nonfat dried milk to minimize non-specific binding. The blots were then incubated with the primary antibody (FXYD11) diluted in 1% BSA and 0.05% sodium azide in PBST and subsequently with the HRP-conjugated secondary antibody diluted in PBST. The blots were developed using the SuperSignal West Pico Detection Kit (#34082, Pierce) and observed in a Universal hood with a cooling-CCD (charge-coupled device) camera (ChemiDoc XRS+, Bio-Rad) and the associated software (Quantity One version 4.6.8, Bio-Rad). Immunoreactive bands were analyzed using Image Lab software version 3.0 (Bio-Rad). The results were converted to numerical values to compare the relative protein abundances of the immunoreactive bands.

To confirm that the immunoreactivity was due to the presence of FXYD11 rather than non-specific binding, various tissue/organ supernatants were subjected to immunoblotting (Figure S1). Meanwhile, rabbit pre-immune serum was substituted for the primary antiserum in the negative control. The immunoblotting protocol was identical to that described above.

### Co-immunoprecipitation (Co-IP)

Gill total lysates were used in this experiment. IP with the FXYD11 antibody, the NKA antibody (α5; positive control), or pre-serum (negative control) was carried out with the ImmunoCruz™ IP/WB Optima System (sc-45042 or sc-45043, Santa Cruz Biotechnology, Santa Cruz, CA, USA) according to the manufacturer's manual. After elution, the samples were stored at 4°C prior to use. To confirm the interaction between FXYD11 and the NKA α-subunit, the above IP solutions were subjected to NKA immunoblotting and analyzed by electrophoresis on a 7% sodium dodecyl sulfate-polyacrylamide gel as described above.

### Whole-mount *in situ* hybridization (WISH) of *fxyd11* in gills

The WISH protocol was modified from Kang et al. [13]. A cDNA fragment corresponding to *fxyd11* (692 bp) of brackish medaka was amplified with Probe-F and R primers (Table S4) by PCR and inserted into the pOSI-T vector (Genemark). After amplifying the target gene by PCR with T7 and SP6 primers, products were subjected to *in vitro* transcription with T7 and SP6 RNA polymerases (Takara), respectively. Dig-labeled RNA probes were examined by RNA gel electrophoresis to confirm their quality and concentration. Four pairs of gills from FW-acclimated brackish medaka were excised and fixed in 4% paraformaldehyde (PFA) in a diethyl pyrocarbonate (DEPC)-PBS solution (1.4 mM NaCl, 0.2 mM KCl, 0.1 mM Na_2_HPO_4_, and 0.002 mM KH_2_PO_4_) at 4°C for 2 h and then washed with DEPC-PBS. The gills were treated with methanol at −20°C overnight and washed with DEPC-PBST (PBS with 0.1% Tween-20) twice for 10 min each. Samples were prehybridized with hybridization buffer (HyB; 60% formamide, 5× SSC (saline-sodium citrate buffer), 0.1% Tween-20, 500 µg/mL yeast tRNA and 50 µg/mL heparin) for 2 h at 65°C and then hybridized with probe (20 ng) in HyB overnight at 67°C. The next day, samples were washed in a series of HyB/SSC solutions at 67°C and then in a series of SSC/PBST buffers at room temperature. Samples were blocked with 5% sheep serum in 2 mg/mL BSA (Sigma-Aldrich, St. Louis, MO, USA) at room temperature for 2 h and then incubated with an anti-Dig Fab (1∶5000 in blocking solution) at 4°C overnight. After washing with DEPC-PBST at room temperature 8 times for 15 min each, gills were exposed to staining buffer (0.1 M Tris pH 9.5, 50 mM MgCl_2_, 0.1 M NaCl and 0.1% Tween-20) twice for 10 min and then developed by incubating with the NBT/BCIP kit (Zymed, South San Francisco, CA, USA) for 1.5 h in the dark. The reaction was stopped by PFA, and the samples were subsequently washed twice with methanol. Samples were stored in DEPC-PBST at 4°C in the dark until further examination and analysis.

### Cryosectioning and immunohistochemical staining

The IHC methods used in this study were modified from previous studies [13], [14], [40]. Gills subjected to *in situ* hybridization were fixed with 10% neutral buffered formalin, infiltrated with OCT (optimal cutting temperature) compound (Sakura, Tissue-Tek, Torrance, CA, USA) overnight at 4°C and then mounted for cryosectioning. The tissue was cryosectioned (5 µm) using a Cryostat Microtome (CM3050S, Leica, Wetzlar, Germany) at −25°C. Sections were placed on 0.01% poly-L-lysine-coated slides (Sigma) and kept in slide boxes at −20°C. Cryosections of *in situ* hybridized gills were washed with PBS and then observed under an optical microscope (BX50, Olympus, Tokyo, Japan). The first micrographs were taken using the cooling-CCD camera (DP72, Olympus) with the associated software (CellSens standard version 1.4, Olympus). Sections were then stained with a monoclonal antibody (α5) to label the NKA α-subunit, followed by a commercial kit (PicTure™; Zymed) for visualizing the immunoreaction. After staining, sections were mounted in Clearmount™ (Zymed), cover-slipped, and the second micrographs were taken in the same region of the same gill filament. Comparing the first and second micrographs of the same region of the gill filament revealed the colocalization of the *in situ* reaction and NKA-immunoreactive (NKA-IR) cells in the gill filaments.

### Statistical analysis

Values are expressed as means ± SEM (standard error of the mean). Results were compared by one-way ANOVA with Tukey's pairwise method using SPSS 12.0 software (SPSS, Inc., Chicago, IL, USA), and *P*<0.05 was set as the significance level.

## Results

### Sequence characteristics and phylogenetic analysis of medaka FXYD proteins

**Figure 1:**
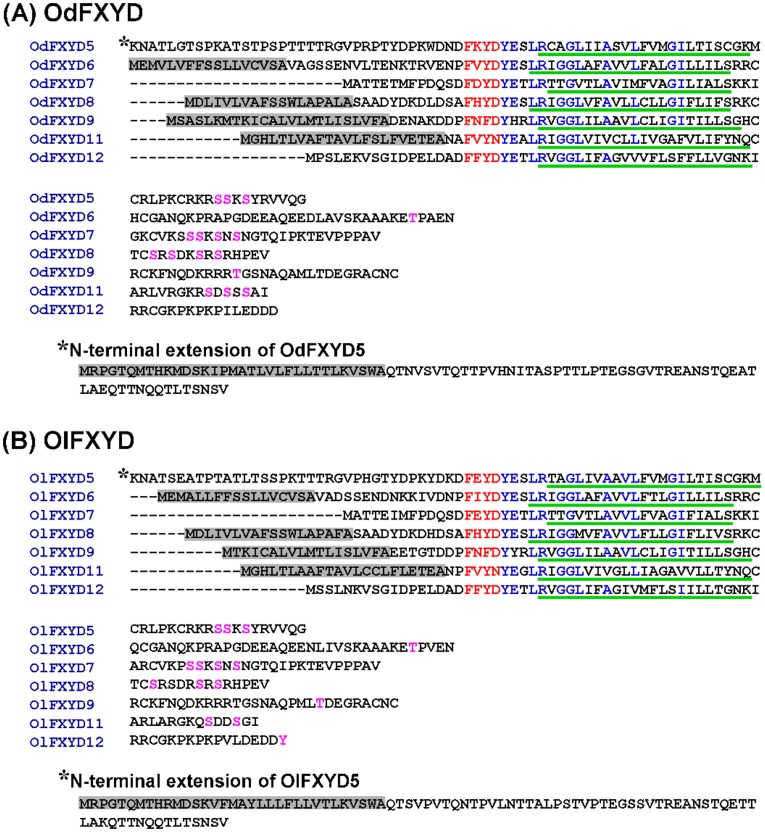
Alignment of putative amino acid sequences of the FXYD proteins in the brackish medaka (Od, A) and Japanese medaka (Ol, B). Red text indicates the FXYD motif; the conserved residues in the conserved region (from the FXYD motif to the end of the transmembrane domain) are shown in blue; the predicted signal peptides are shown with a gray background; the predicted transmembrane domains are shown in green underlined text; and the predicted sites for phosphorylation are shown in pink text.

**Figure 2:**
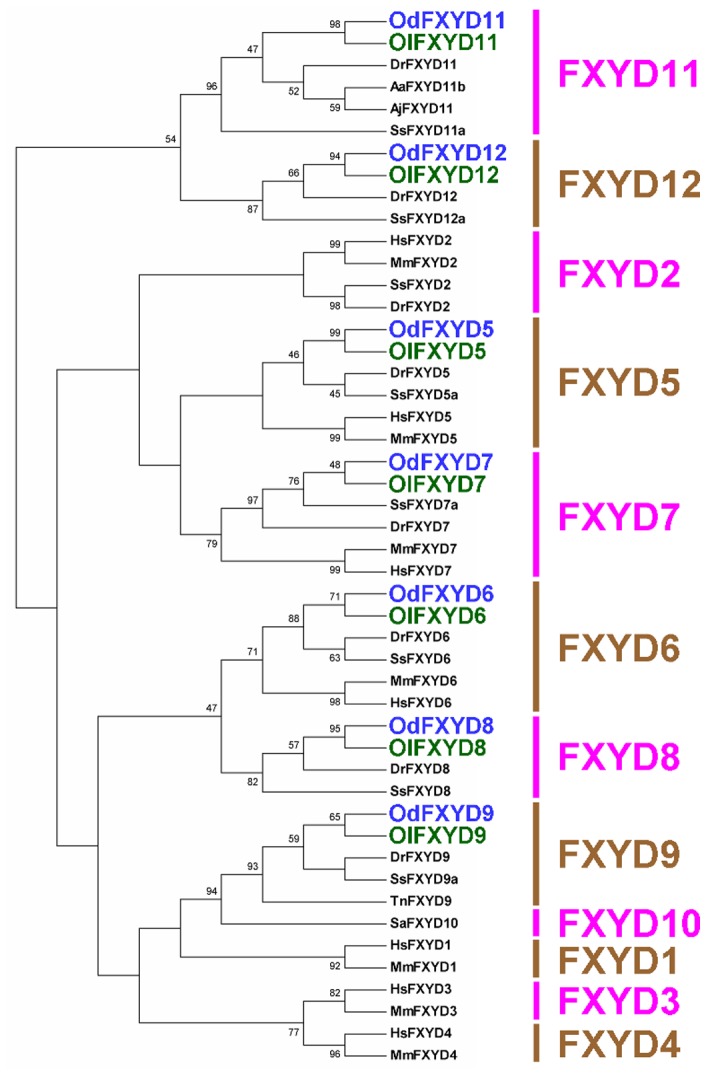
Phylogenetic tree of FXYD proteins using the minimum evolution method. The tree was generated using FXYD protein sequences from different species, including human (Hs, *Homo sapiens*), mouse (Mm, *Mus musculus*), spiny dogfish (Sa, *Squalus acanthias*), zebrafish (Dr, *Danio rerio*), Atlantic salmon (Ss, *Salmo salar*), European eel (Aa, *Anguilla anguilla*), Japanese eel (Aj, *Anguilla japonica*), brackish medaka (Od, *Oryzias dancena*), and Japanese medaka (Ol, *O. latipes*). Numbers represent bootstrap values for the percentage of 1000 replicates. Accession numbers are listed in Tables S1 and S3.

Fourteen members of the FXYD protein family were cloned from brackish medaka (OdFXYD) and Japanese medaka (OlFXYD)(Figure 1; accession numbers are listed in Table S1). All fourteen were small proteins, comprising approximately 65–176 amino acid residues (Table S2). An alignment revealed that these FXYD proteins contain the highly similar FXYD motif followed by a tyrosine and a number of conserved residues in the transmembrane domain (red and blue text in Figure 1, respectively). The deduced protein sequences were examined for the potential presence of signal peptides, membrane domains, phosphorylation sites, and glycosylation sites (details are provided in Table S2). A hydropathy analysis determined that all of the medaka FXYD proteins contain one transmembrane domain (green underlined text in Figure 1), while most also contain a signal peptide (gray background) and phosphorylation sites (pink text)(Figure 1; Table S2). Forty-eight FXYD proteins from ten vertebrate species, including fish and mammals, were then aligned and compared (Figure 2; accession numbers are listed in Table S3). The teleostean FXYD11 and 12 proteins were isolated from other members of the FXYD protein family in the phylogenetic tree. Across all FXYD family members, the FXYD proteins of the two medaka species were very closely related and clearly grouped in the same clade. Moreover, higher identities were found between the homologous FXYD proteins of the two medaka species as compared with the other FXYD homologues from teleosts and mammals (Table 1). Among the different FXYD members, teleost FXYD9 proteins showed the highest identity (90.0–97.5%).

**Table 1:** Amino acid identity (%) of FXYD proteins of the brackish medaka and Japanese medaka as compared with other homologues from teleosts and mammals.

Medaka	Teleosts				Mammals	
	OlFXYD	DrFXYD	SsFXYD	OnFXYD	HsFXYD	MmFXYD
Brackish medaka (Od)						
OdFXYD5	87.5	60.0	55.0	—	42.5	35.0
OdFXYD6	90.0	82.5	65.0	87.5	72.5	72.5
OdFXYD7	85.0	72.5	70.0	87.5	47.5	52.5
OdFXYD8	90.0	67.5	70.0	72.5	—	—
OdFXYD9	97.5	95.0	92.5	97.5	—	—
OdFXYD11	80.0	55.0	50.0	50.0	—	—
OdFXYD12	87.5	82.5	62.5	80.0	—	—
Japanese medaka (Ol)						
OlFXYD5	—	60.0	57.5	—	47.5	37.5
OlFXYD6	—	80.0	60.0	90.0	72.5	72.5
OlFXYD7	—	70.0	70.0	80.0	45.0	47.5
OlFXYD8	—	75.0	75.0	77.5	—	—
OlFXYD9	—	92.5	90.0	95.0	—	—
OlFXYD11	—	60.0	60.0	52.5	—	—
OlFXYD12	—	80.0	62.5	80.0	—	—

Homologies were evaluated for the 40 amino acids of the conserved region (from 3 amino acids before the FXYD motif to the rear of the transmembrane domain). Accession numbers are listed in Tables S1 and S3. Dr, zebrafish; Ss, Atlantic salmon; On, Nile tilapia; Hs, human; Mm, mouse; —, not applicable.

### Tissue distribution of *fxyd* genes

**Figure 3:**
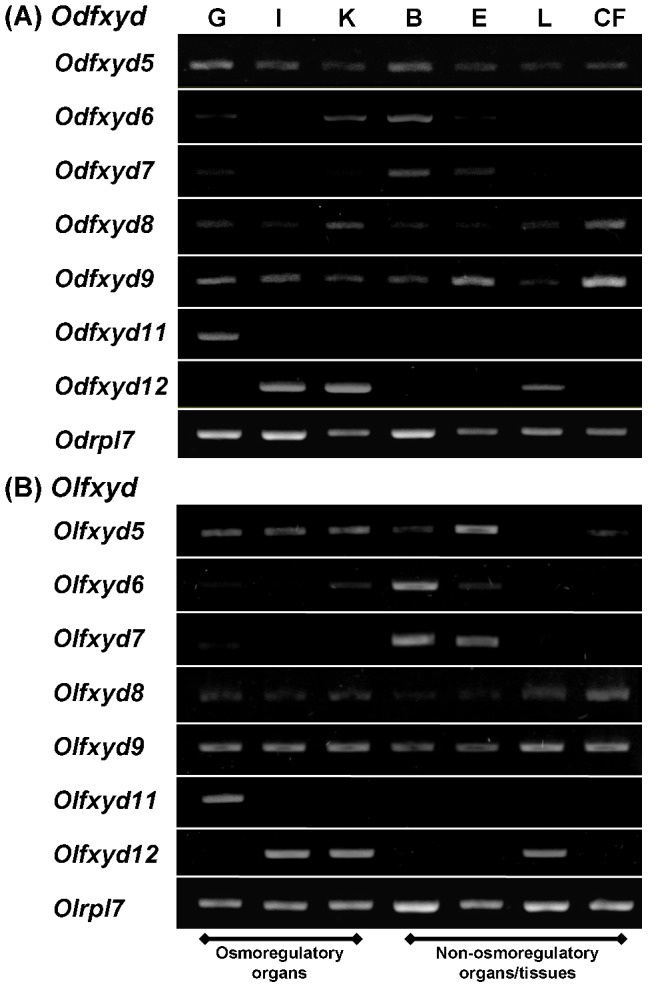
Tissue distribution of *fxyd* genes as detected by RT-PCR in the brackish water-acclimated brackish medaka (Od, A) and freshwater-acclimated Japanese medaka (Ol, B). The *rpl7* gene was used as an internal control. G, gill; I, intestine; K, kidney; B, brain; E, eye; L, liver; CF, caudal fin.

An RT-PCR analysis followed by electrophoresis characterized the tissue-specific expression pattern of *fxyd* genes in the BW-acclimated brackish medaka and FW-acclimated Japanese medaka (Figure 3). The *rpl7* gene was used as an internal control to confirm cDNA quality. Expression profiles of the 14 *fxyd* genes were analyzed in the osmoregulatory organs (gill, kidney, and intestine) as well as other organs/tissues. Similar patterns were observed in the brackish medaka and Japanese medaka. Most of the *fxyd* genes were expressed in the osmoregulatory organs, among which three *fxyd* genes (*fxyd5*, *8*, and *9*) were broadly distributed in a number of different organs/tissues, and *fxyd11* was expressed most prominently in the gill.

### Branchial *fxyd* mRNA levels

**Figure 4:**
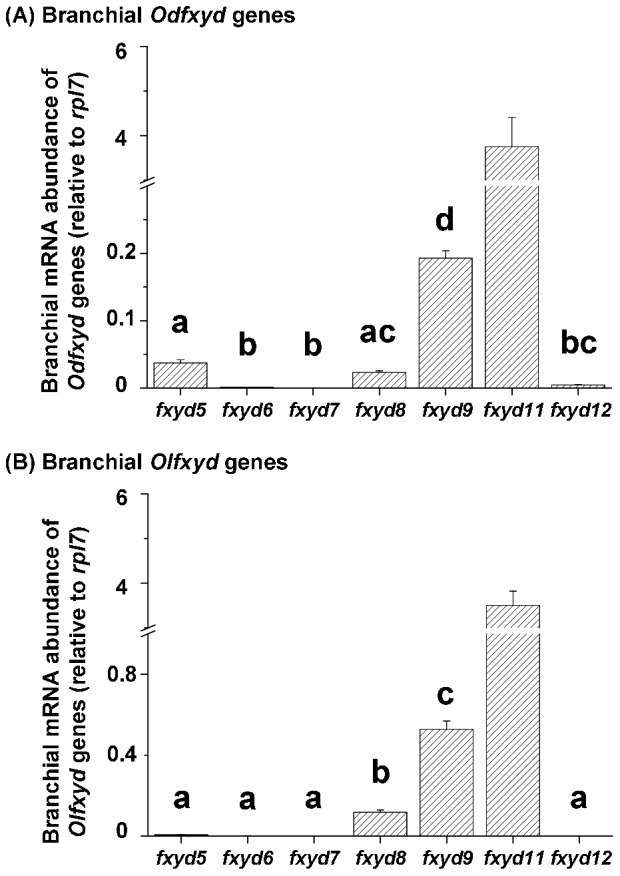
Comparisons of branchial *fxyd* mRNA abundance in the brackish medaka (Od, A) and the Japanese medaka (Ol, B). Values are means ± SEM (N = 10). Different letters indicate significant differences among *fxyd* genes, excluding *fxyd11* (one-way ANOVA with Tukey's comparison, *P*<0.05).

The expression profiles of branchial *fxyd* genes were similar between the brackish medaka and Japanese medaka (Figure 4). In the gill, *fxyd11* and *fxyd9* mRNA levels were the highest and second highest, respectively, among all detected *fxyd* genes in both medaka species. The gill mRNA levels of *fxyd11* were approximately 19- and 7-fold higher than that of *fxyd9* in brackish medaka and Japanese medaka, respectively. Conversely, the mRNA levels of *fxyd6*, *7*, and *12* were low in the gills of both medaka species.

### Salinity effects on the expression of branchial *fxyd* genes

**Figure 5:**
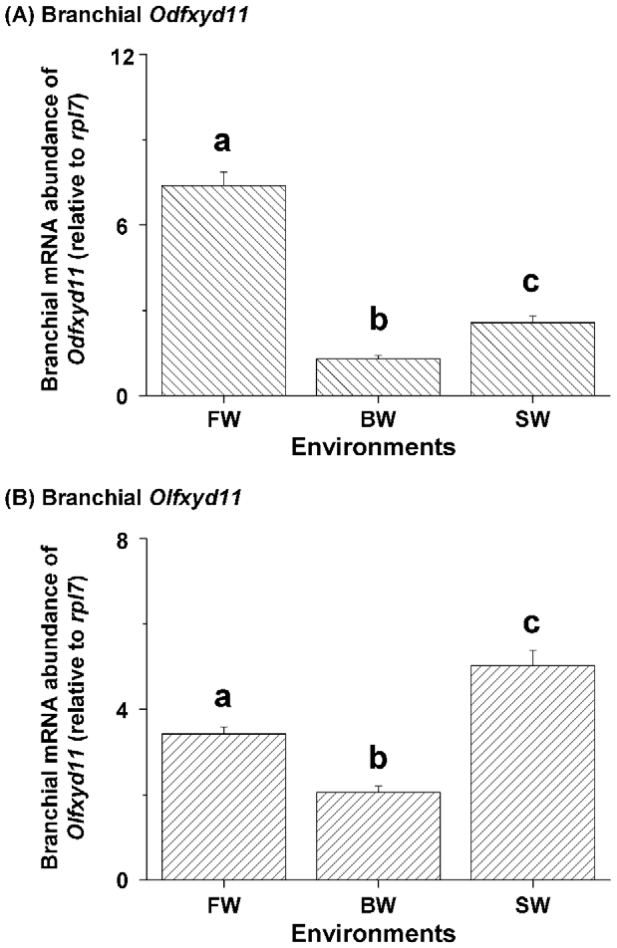
Salinity effects on branchial *fxyd11* mRNA abundance in the brackish medaka (Od, A) and the Japanese medaka (Ol, B). Values are means ± SEM (N = 6). Different letters indicate significant differences among salinity groups (one-way ANOVA with Tukey's comparison, *P*<0.05). FW, fresh water; BW, brackish water; SW, seawater.

**Figure 6:**
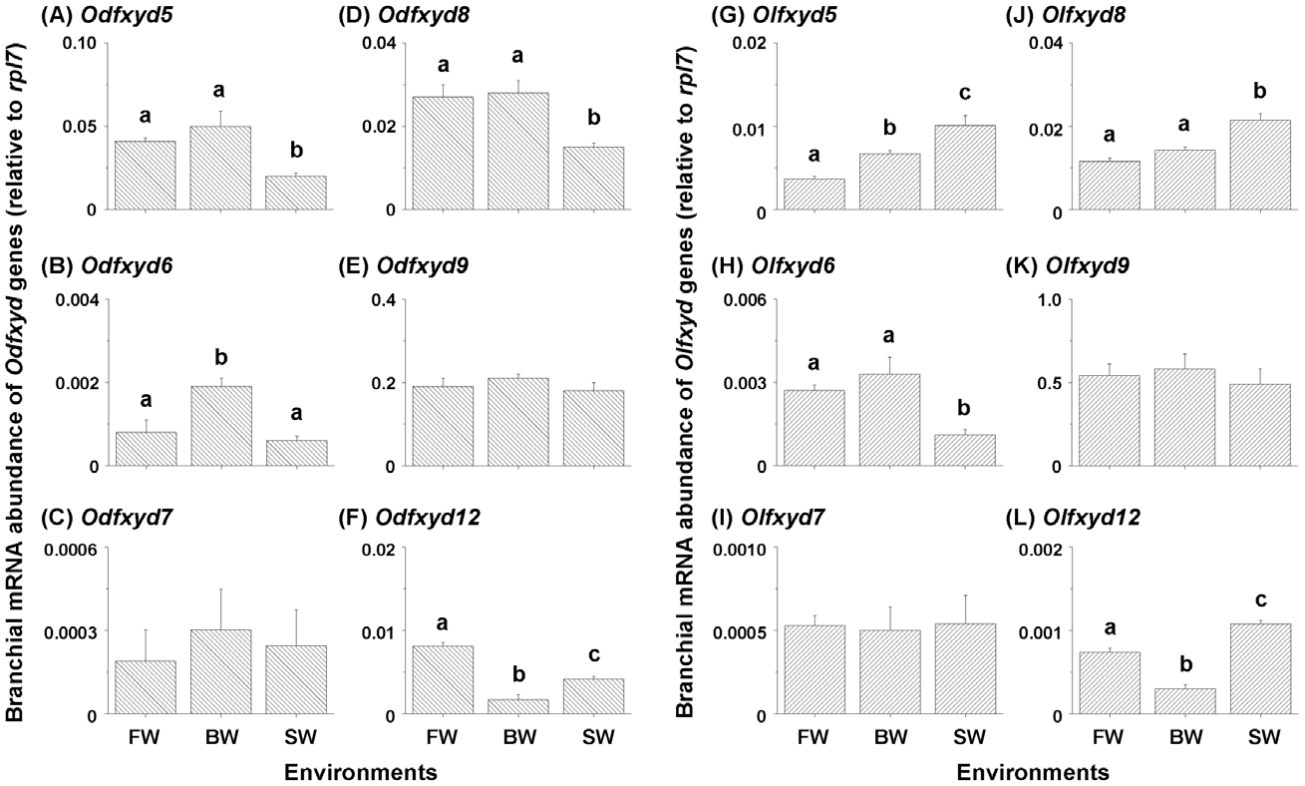
Salinity effects on multiple *fxyd* mRNA abundance in gills of the brackish medaka (Od, A–F) and Japanese medaka (Ol, G–L). Values are means ± SEM (N = 4). Different letters indicate significant differences among salinity groups (one-way ANOVA with Tukey's comparison, *P*<0.05). FW, fresh water; BW, brackish water; SW, seawater.

The expression of most *fxyd* genes in the gills of brackish medaka and Japanese medaka was salinity-dependent (Figures 5 and 6). Gill *Odfxyd11* mRNA levels were significantly higher in FW-acclimated brackish medaka than in BW- or SW-acclimated fish (approximately 5.7-fold; Figure 5A; Table 2). In the Japanese medaka, however, the highest relative mRNA abundance of branchial *Olfxyd11* was found in the SW-acclimated individuals (Figure 5B). Figure 6 shows the expression levels of other *fxyd* genes in medaka acclimated to FW, BW, and SW. The expression pattern of *Odfxyd12* was similar to that of *Odfxyd11* in the gills of the brackish medaka (Figure 6F). The highest levels of branchial *Odfxyd5*, *6*, and *8*, however, were found in the gills of BW-acclimated brackish medaka (Figure 6A, B, D). No significant difference in branchial *Odfxyd7* and *9* expression was found among the salinity groups (Figure 6C and E). In contrast, the mRNA levels of *Olfxyd5*, *8*, and *12* were significantly higher in gills of SW-acclimated Japanese medaka than in the other salinity groups (Figure 6G, J, and L). Similar to the branchial *fxyd* patterns of the brackish medaka, the highest level of *Olfxyd6* was found in the gills of BW-acclimated Japanese medaka (Figure 6H), and the expression of *Olfxyd7* and *9* was not salinity-dependent (Figure 6I and K).

**Table 2:** Salinity effects on FXYD11 and NKA expression patterns in gills of the brackish medaka (Od) and Japanese medaka (Ol).

Medaka	Protein	Expression ratios (FW ∶ BW ∶ SW)
Od	mRNA levels	
	FXYD11	5.7^a^ ∶ 1.0^b^ ∶ 2.0^c^
	NKA α-subunit	3.7^a^ ∶ 1.0^b^ ∶ 1.3^b^
	Protein abundance	
	FXYD11	2.0^a^ ∶ 1.0^b^ ∶ 1.3^b^
	NKA α-subunit	1.6^a^ ∶ 1.0^b^ ∶ 1.7^a^
	Activity	
	NKA	4.5^a^ ∶ 1.0^b^ ∶ 3.7^a^
Ol	mRNA levels	
	FXYD11	1.0^a^ ∶ 0.6^b^ ∶ 1.5^c^
	NKA α-subunit	1.0^a^ ∶ 0.8^a^ ∶ 1.6^b^
	Protein abundance	
	FXYD11	—
	NKA α-subunit	1.0^a^ ∶ 2.3^b^ ∶ 2.5^b^
	Activity	
	NKA	1.0^a^ ∶ 1.4^a^ ∶ 3.9^b^

The ratios were calculated based on the expression of the brackish water-acclimated brackish medaka or freshwater-acclimated Japanese medaka. The NKA expression data were modified from Kang et al. [12]. Different letters indicate significant differences among salinity groups (one-way ANOVA with Tukey's comparison, *P*<0.05). FW, fresh water; BW, brackish water; SW, seawater; —, not determined.

### Expression patterns of gill-specific FXYD11 in the brackish medaka

**Figure 7:**
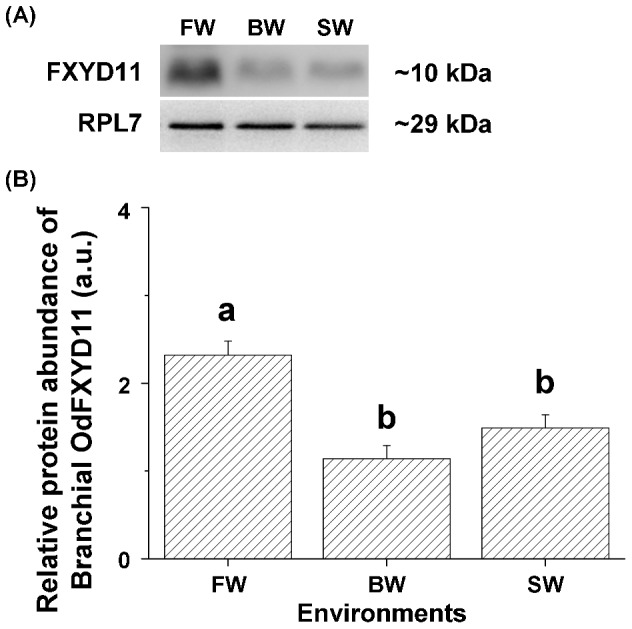
Salinity effects on branchial OdFXYD11 protein abundance in the brackish medaka. Representative immunoblots (A) and relative intensities (B) of OdFXYD11 and OdRPL7 (internal control) expression in the various groups. Values are means ± SEM (N = 5). Different letters indicate significant differences among salinity groups (one-way ANOVA with Tukey's comparison, *P*<0.05). FW, fresh water; BW, brackish water; SW, seawater; a.u., arbitrary units.

**Figure 8:**
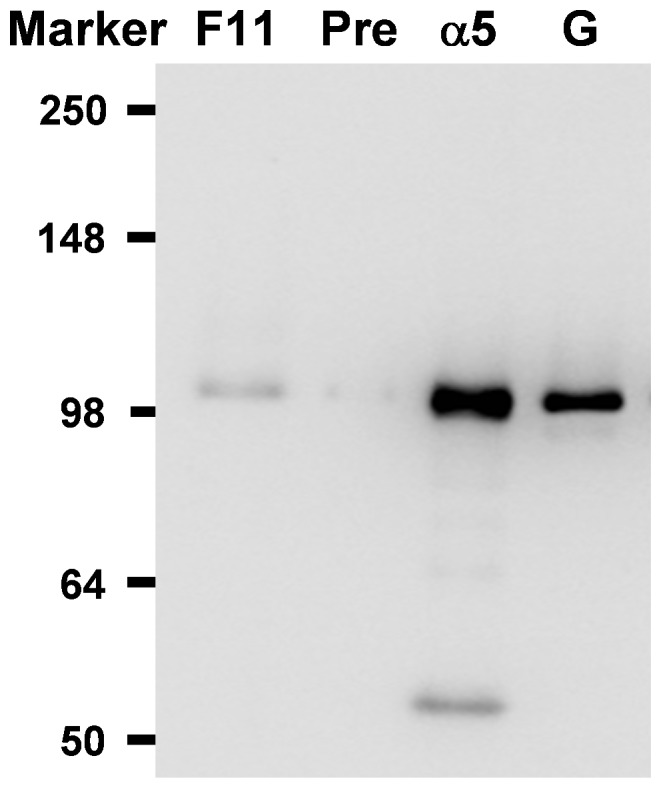
Co-immunoprecipitation of OdNKA with OdFXYD11 in freshwater-acclimated brackish medaka. FXYD11 was immunoprecipitated from gill total lysates with a primary antibody, and then the immune complexes were analyzed by SDS-PAGE and immunoblotted for the NKA protein. Immunoreactive bands for NKA were detected at 100 kDa. F11, western blot detection of the FXYD11 antibody (experimental group); Pre, negative immunoblot control using pre-immune serum for the immunoprecipitation; α5, positive immunoblot control using the same antibody (NKA, α5) for the immunoprecipitation; G, positive immunoblot control using gill total lysates without the immunoprecipitation.

Quantification of the immunoreactive bands in protein samples from different salinity groups revealed that FW-acclimated brackish medaka had the highest abundance of branchial OdFXYD11, 2.0-fold higher than the levels detected in the other groups (Figure 7; Table 2). The interaction between OdFXYD11 and OdNKA was then examined by co-immunoprecipitation (Co-IP; Figure 8). When FXYD11 was precipitated, a immunoreactive band was detected at 100 kDa (F11, lane 1 of Figure 8), corresponding to the molecular mass of the NKA α-subunit protein. No Co-IP immunoreaction was observed following the use of pre-immune serum (Pre, lane 2 of Figure 8). Lane 3 (α5; IP with the NKA antibody) and lane 4 (G; gill total lysates without IP) are positive controls demonstrating the immunoprecipitation efficiency. These data indicate that OdFXYD11 possibly interacts with NKA in the gills of the brackish medaka.

**Figure 9:**
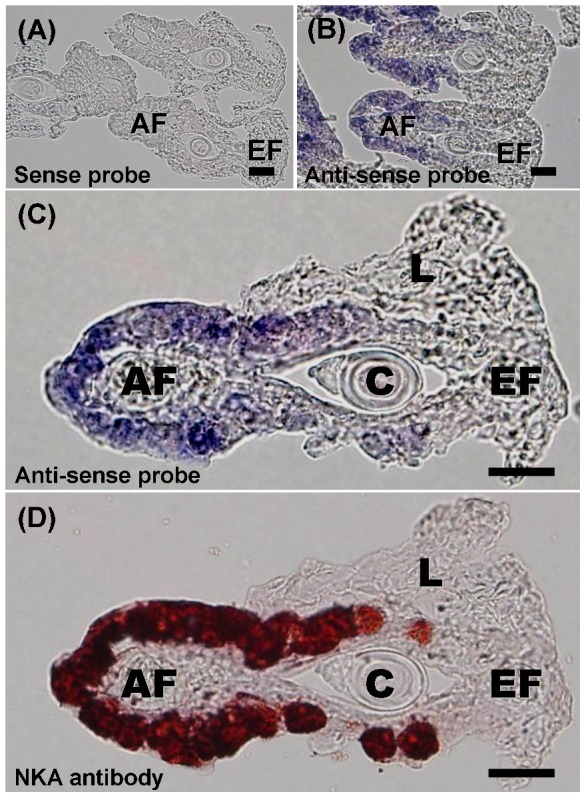
Localization of *Odfxyd11* mRNA in gill sections of freshwater-acclimated brackish medaka. Cryosections of gill filaments labeled with sense probe (A; control) or anti-sense probe (B) show counterstaining with *Odfxyd11* probes. (C) Positive *Odfxyd11* signals (purple) were localized to epithelial cells of the gill filament. (D) The NKA immunoreactive cells (dark red) are identical to the epithelial cells labeled with the *Odfxyd11* probe in the same cryosection. AF, afferent region; C, cartilage; EF, efferent region; L, lamellae. Scale bar = 20 µm.

To determine the localization pattern of *Odfxyd11*, an anti-sense RNA probe was used to detect *Odfxyd11* in gills of the FW-acclimated brackish medaka (Figure 9). Positive signals (purple) were mainly confined to the epithelial cells of the afferent region of the gill filament (Figure 9B and C). The sense probe revealed no signal in the same region (Figure 9A). Gill cryosections were then counterstained with the NKA α-subunit antibody (α5), which revealed that the *Odfxyd11*-expressing epithelial cells also showed immunoreactivity for NKA (Figure 9D). Taken together, these results demonstrate that *Odfxyd11* expression is localized to the NKA-IR cells in the gill epithelia of the brackish medaka and that OdFXYD11 possibly interacts with the NKA α-subunit upon salinity challenge.

## Discussion

In teleosts, branchial NKA proteins play a crucial role in osmoregulatory organs in driving the action of other transporters and channels to regulate ion concentrations and maintain a stable internal environment [16], [21]. It has recently become clear that mammalian FXYD proteins modulate NKA activity [24], [26]–[28]. To date, few studies have investigated the salinity-dependent expression of FXYD proteins in teleosts, and these studies have focused on a rather limited group of species, including the euryhaline pufferfish [32] and tilapia [37], the anadromous salmon [34]–[36], the catadromous eel [38], and the stenohaline zebrafish [33]. In this study, we revealed similar and different expression of branchial *fxyd* genes in two closely related medaka species. The results will shed more light on the relationship between FXYD proteins and NKA of euryhaline teleosts. The present study identified novel members of the teleostean FXYD protein family and examined their tissue distributions and responses to changes in environmental salinity. We also investigated the effects of salinity on the protein abundance of gill-specific FXYD11 in the brackish medaka. Our results indicate that the brackish medaka is an appropriate and useful saltwater (including BW and SW) fish model for osmoregulatory studies.

Fourteen members of FXYD protein family (FXYD5–9, 11, and 12) were identified in the brackish medaka and Japanese medaka (Figures 1 and 2). According to the deduced amino acid sequences, these medaka FXYD proteins share the characteristic features of other FXYD proteins in mammals and fish, including one transmembrane domain with an extracellular N-terminus, a cytoplasmic C-terminus, and a highly similar FXYD motif at the N-terminus (Figure 1). Moreover, the high degree of homology between the FXYD proteins of the two species (Figure 2; Table 1) not only further strengthens their close phylogenetic relationship but also implies that the homologous FXYD proteins might serve similar functions [32], [35]. To date, eight members of the FXYD protein family (FXYD2, 5–9, 11, and 12) have been reported in both the Atlantic salmon and the zebrafish [33], [34]. Relative to mammalian FXYD proteins, FXYD9–12 are specific to fish. However, unlike the FXYD proteins of mammals and sharks, the functions of the teleost FXYD proteins are poorly understood. Mammalian and shark FXYD proteins were reported to function as modulators of NKA, with different family members exerting different effects on NKA activity [24], [27], [28]. For instance, FXYD1 and FXYD10 were shown to inhibit NKA activity [30], [49], whereas FXYD4 was reported to stimulate NKA activity [50]. Although the functions and molecular mechanisms of teleostean FXYD proteins have not yet been clarified, additional studies aimed at understanding the effects of salinity on teleost FXYD protein expression profiles will likely illustrate the potential roles of FXYD proteins in the osmoregulatory response to salinity challenges.

Most medaka *fxyd* genes were expressed in osmoregulatory organs (i.e., gill, kidney, and intestine; Figure 3). Similar patterns were reported in the Atlantic salmon and the zebrafish [33], [34]. Like the mammalian *fxyd* genes [24], [27], the expression of certain *fxyd* genes was tissue-specific in both medaka species. Similar to rat *fxyd7* [51], *fxyd7* is primarily expressed in the brain of the brackish medaka, Japanese medaka (Figure 3), and Atlantic salmon [34]. However, zebrafish *fxyd7* was ubiquitously distributed [33]. In contrast, medaka *fxyd5*, *8*, and *9* were broadly distributed in various organs/tissues. Similar patterns were observed for *fxyd8* in the zebrafish and for *fxyd9* in all reported teleosts [32]–[34], [37]. Conversely, in the Atlantic salmon, *fxyd5* is predominantly expressed in the kidney and heart [34]. Although the tissue distributions of teleost *fxyd* genes thus vary across species, the patterns were very similar between the brackish medaka and Japanese medaka. For example, *fxyd11* was highly enriched in the gills of both medaka species (Figures 3 and [4]) as well as the other studied teleosts, with the exception of the spotted green pufferfish [32]–[34],[37],[38]. FXYD11 interacts with the NKA α-subunit in branchial NKA-IR cells of the brackish medaka (Figures 8 and [9]), the zebrafish [33], and the Atlantic salmon [35]. These interactions have been established through different approaches, including Co-IP, *in situ* hybridization, *in situ* proximity ligation assay, and immunostaining.

Among all detected *fxyd* genes, *fxyd11* was the most highly expressed in the gills of the brackish medaka, Japanese medaka (Figure 4), and Atlantic salmon [34]. In addition, higher levels (mRNA/protein) of branchial FXYD11 were found in SW-acclimated Atlantic salmon and Japanese eel (*Anguilla japonica*) as well as in ion-poor water-acclimated zebrafish relative to FW-acclimated individuals [33], [35], [38]. These studies also showed that the expression of FXYD11 and the NKA α-subunit increased in parallel [16], indicating that teleostean FXYD11 might enhance NKA activity in the gills [33], [35].

Salinity-dependent expression of branchial *fxyd11* was also observed in the brackish medaka and Japanese medaka (Figure 5). Different from the comparisons made between FW and SW groups in the other studied teleosts, we acclimated individuals from the two medaka species to FW, BW, or SW for subsequent investigations. The expression patterns (U-shape) of branchial *fxyd11* were similar between the two species, but the highest expression was found in different salinity groups (Figure 5). Similar to the other studied teleosts, in the Japanese medaka, *Olfxyd11* was more highly expressed in SW-acclimated individuals than in FW-acclimated fish [35], [38]. Conversely, higher branchial *Odfxyd11* expression was detected in FW-acclimated brackish medaka. When the branchial mRNA levels of *Olfxyd11* were compared to those of the *nka* α-subunit in the Japanese medaka, parallel increases in expression were noted (Table 2), similar to other teleosts [35], [38]. In the gills of the brackish medaka, however, the OdFXYD11 expression patterns (both mRNA and protein abundance) differed from those of the NKA α-subunit (Table 2), such that the expression of branchial OdFXYD11 did not change with NKA expression. Possible reasons may be more than one, such as the composition of NKA α-subunits isoforms or other functions of FXYD11. Although further mechanism is yet unknown, this is the first case of uncorrelated FXYD11/NKA expression to be reported in the teleost. Furthermore, previous time-course experiments revealed that the mRNA abundance of branchial *fxyd11* was not correlated with branchial NKA expression (either mRNA or activity) in the Atlantic salmon and the Mozambique tilapia [35]–[37]. On the other hand, mRNA and protein expression of branchial FXYD11 was found to be influenced by hormones [35], [37], [38]. These results indicate that FXYD11 modulates NKA activity in multiple ways rather than simply increasing expression in parallel with NKA. In mammals, FXYD proteins affect NKA responses (e.g., composition, apparent ion affinities, and maximal pump current) via differential phosphorylation, hormones, and interactive levels [18], [27], [52]. Taken together, these observations on the distribution and expression of FXYD11 in the two medaka species indicate that FXYD11 is a unique member of the FXYD protein family specific to the gill and that branchial FXYD11 may play a crucial role in osmoregulation via modulating NKA expression [33], [34], [38]. Our results also suggest that FXYD11 might use diverse modulatory mechanisms in response to salinity challenges in the gills of the two medaka species.

In the gills of both the brackish medaka and Japanese medaka, *fxyd9* was the second most highly expressed *fxyd* gene, but its expression was not salinity-dependent (Figures 4 and 6). This pattern is different from that of the spotted green pufferfish, where higher levels (mRNA and protein) of branchial FXYD9 were found in FW-acclimated individuals as compared with SW-acclimated fish [32]. In time-course experiments performed with the Atlantic salmon, however, the expression of branchial *fxyd9* showed no differences between time-points after the fish were transferred from FW to SW and *vice versa* [35], [36]. In the gills of the Mozambique tilapia, *fxyd9* levels increased when the fish were transferred from SW to FW and rose quickly and then recovered gradually upon transfer from FW to SW [37]. Although the expression patterns of branchial *fxyd9* differ among these teleosts, the higher levels of branchial FXYD9 in the spotted green pufferfish and Mozambique tilapia were found in environments of low NKA activity [32], [37]. In the spotted green pufferfish, branchial FXYD9 was inferred to inhibit NKA activity because of its high similarity to human FXYD3 and shark FXYD10 as well as the opposite expression pattern of NKA after salinity challenges [32]. In this study, the constant and broadly distributed expression of *fxyd9* suggests that it may function as a housekeeping gene and play a role in co-modulating NKA expression with FXYD11 in the gills of the two medaka species. The expression levels of the other branchial *fxyd* genes were much lower, especially *fxyd6*, *7*, and *12*, relative to *fxyd11* and *9* (Figure 4). Although the gill expression of several *fxyd* genes (i.e., *fxyd5*, *6*, *8*, and *12*) was salinity-dependent in both species (Figure 6), the functions of these genes require further elucidation.

In this study, we investigated changes in the expression of the *fxyd* genes in two closely related medaka species upon salinity challenges. Our comparison of *fxyd* genes expression patterns between two medaka species with diverse osmoregulatory capabilities revealed both similar (e.g., tissue distributions) and different (e.g., *fxyd11* expression) patterns. Moreover, a different pattern was found that the branchial OdFXYD11 expression was the highest in the FW-acclimated individuals, unlike those reported in the other teleosts including the Japanese medaka (Figure 5) [35], [38]. These results indicate that the species' responses (e.g., FXYD and NKA expression and salinity tolerance) to salinity changes might be related to their evolutionary histories, natural habitats, and/or developmental stages [22], [53], [54]. Thus, the choice of an appropriate animal model is important for osmoregulatory studies involving salinity challenges. Based on their close phylogenetic relationships and diverse characteristics, *Oryzias* species offer unique models for comparative and osmoregulatory studies [3], [4], [55]. The brackish medaka represents a unique model because it shows better salinity tolerance and hyposmoregulatory ability than the Japanese medaka [3], [4],[12]. Moreover, the entire genome of the Japanese medaka has been sequenced and annotated, thus facilitating molecular studies of the closely related brackish medaka, such as the cloning, mRNA expression, and *in situ* hybridizations performed in this study. The brackish medaka is therefore an applicable saltwater (including BW and SW) fish model for osmoregulatory studies.

## Conclusions

The present study revealed that the protein sequences and tissue distributions of FXYD proteins were very similar in the closely related brackish medaka and Japanese medaka species. Upon salinity challenge, however, the expression profiles of most branchial *fxyd* genes differed between the two species. Among the branchial *fxyd* genes, *fxyd11* was highly and specifically expressed in the gills of both medaka species. The expression pattern of branchial *Olfxyd11* was similar to that of the *nka* α-subunit in the Japanese medaka, but non-correlated expression patterns were observed for OdFXYD11 and NKA in the brackish medaka at both the mRNA and protein levels. This finding (non-correlated expression patterns) is the first report of teleost FXYD proteins in a chronic (i.e., acclimated) rather than an acute (i.e., time-course) salinity challenge experiment. Although the brackish medaka and Japanese medaka are very closely related, their branchial FXYD proteins exhibit divergent expression patterns. To our knowledge, this report is the first to focus on FXYD expression in the gills of closely related euryhaline teleosts. Given the sophisticated tools available for the Japanese medaka system, the closely related brackish medaka is highly recommended as a saltwater fish model for osmoregulatory studies.

## Supporting Information

Figure S1 Representative immunoblots of FXYD11 and RPL7 in the brackish medaka. (A) Immunoblots of various tissues from freshwater-acclimated brackish medaka showed that FXYD11 (arrow) was only expressed in the gill (G) and had a molecular mass of approximately 10 kDa. (B) Pre-immune serum was used as the negative control for FXYD11. (C) The representative immunoblot of PRL7 in the gills. M, marker (kDa); K, kidney; I, intestine; E, eye; L, liver; Go, gonad; Mu, muscle; FG, the gill from freshwater-acclimated individuals; BG, the gill from brackish water-acclimated individuals; SG, the gill from seawater-acclimated individuals.
(TIF)

Table S1 Accession numbers of FXYD proteins from the brackish medaka and Japanese medaka.
(DOC)

Table S2 Predicted sequence information for FXYD proteins from the brackish medaka and Japanese medaka.
(DOC)

Table S3 FXYD protein accession numbers.
(DOC)

Table S4 Primer sequences and probe construction used for RACE, RT-PCR, and WISH of *fxyd* genes of the brackish medaka (Od) and Japanese medaka (Ol).
(DOC)

Table S5 Probe construction used for Q-PCR of *fxyd* genes of the brackish medaka and Japanese medaka.
(DOC)

## References

[1] Roberts TR (1998) Systematic observations on tropical Asian medakas or ricefishes of the genus *Oryzias* with descriptions of four new species. Ichthyol Res 45: 213–224.

[2] Parenti LR (2008) A phylogenetic analysis and taxonomic revision of ricefishes, *Oryzias* and relatives (Beloniformes, Adrianichthyidae). Zool J Linn Soc 154: 494–610.

[3] Inoue K, Takei Y (2002) Diverse adaptability in *Oryzias* species to high environmental salinity. Zool Sci 19: 727–734. 12149572 10.2108/zsj.19.727

[4] Inoue K, Takei Y (2003) Asian medaka fishes offer new models for studying mechanisms of seawater adaptation. Comp Biochem Physiol B Biochem Mol Biol 136: 635–645. 14662290 10.1016/s1096-4959(03)00204-5

[5] Yusof S, Ismail A, Koito T, Kinoshita M, Inoue K (2012) Occurrence of two closely related ricefishes, Javanese medaka (*Oryzias javanicus*) and Indian medaka (*O. dancena*) at sites with different salinity in Peninsular Malaysia. Environ Biol Fish 93: 43–49.

[6] Sakamoto T, Kozaka T, Takahashi A, Kawauchi H, Ando M (2001) Medaka (*Oryzias latipes*) as a model for hypoosmoregulation of euryhaline fishes. Aquaculture 193: 347–354.

[7] Kinoshita M, Murata K, Naruse K, Tanaka M (2009) Medaka: Biology, Management, and Experimental Protocols. Ames: Wiley-Blackwell Press.

[8] Chen X, Li L, Wong CK, Cheng SH (2009) Rapid adaptation of molecular resources from zebrafish and medaka to develop an estuarine/marine model. Comp Biochem Physiol C Toxicol Pharmacol 149: 647–655. 19302835 10.1016/j.cbpc.2009.01.009

[9] Sasado T, Tanaka M, Kobayashi K, Sato T, Sakaizumi M, et al (2010) The National BioResource Project Medaka (NBRP Medaka): an integrated bioresource for biological and biomedical sciences. Exp Anim 59: 13–23. 20224166 10.1538/expanim.59.13

[10] Kasahara M, Naruse K, Sasaki S, Nakatani Y, Qu W, et al (2007) The medaka draft genome and insights into vertebrate genome evolution. Nature 447: 714–719. 17554307 10.1038/nature05846

[11] Wittbrodt J, Shima A, Schartl M (2002) Medaka–a model organism from the far East. Nat Rev Genet 3: 53–64. 11823791 10.1038/nrg704

[12] Kang CK, Tsai SC, Lee TH, Hwang PP (2008) Differential expression of branchial Na^+^/K^+^-ATPase of two medaka species, *Oryzias latipes* and *Oryzias dancena*, with different salinity tolerances acclimated to fresh water, brackish water and seawater. Comp Biochem Physiol A Mol Integr Physiol 151: 566–575. 18692588 10.1016/j.cbpa.2008.07.020

[13] Kang CK, Tsai HJ, Liu CC, Lee TH, Hwang PP (2010) Salinity-dependent expression of a Na^+^, K^+^, 2Cl^−^ cotransporter in gills of the brackish medaka *Oryzias dancena*: a molecular correlate for hyposmoregulatory endurance. Comp Biochem Physiol A Mol Integr Physiol 157: 7–18. 20576485 10.1016/j.cbpa.2010.05.013

[14] Kang CK, Yang WK, Lin ST, Liu CC, Lin HM, et al (2013) The acute and regulatory phases of time-course changes in gill mitochondrion-rich cells of seawater-acclimated medaka (*Oryzias dancena*) when exposed to hypoosmotic environments. Comp Biochem Physiol A Mol Integr Physiol 164: 181–191. 22960413 10.1016/j.cbpa.2012.08.010

[15] Hirose S, Kaneko T, Naito N, Takeib Y (2003) Molecular biology of major components of chloride cells. Comp Biochem Physiol B Biochem Mol Biol 136: 593–620. 14662288 10.1016/s1096-4959(03)00287-2

[16] Hwang PP, Lee TH, Lin LY (2011) Ion regulation in fish gills: recent progress in the cellular and molecular mechanisms. Am J Physiol Regul Integr Comp Physiol 301: R28–R47. 21451143 10.1152/ajpregu.00047.2011

[17] Scheiner-Bobis G (2002) The sodium pump. Its molecular properties and mechanics of ion transport. Eur J Biochem 269: 2424–2433. 12027879 10.1046/j.1432-1033.2002.02909.x

[18] Toyoshima C, Kanai R, Cornelius F (2011) First crystal structures of Na^+^,K^+^-ATPase: new light on the oldest ion pump. Structure 19: 1732–1738. 22153495 10.1016/j.str.2011.10.016

[19] Cornelius F, Mahmmoud YA (2003) Functional modulation of the sodium pump: the regulatory proteins “Fixit”. News Physiol Sci 18: 119–124. 12750449 10.1152/nips.01434.2003

[20] Aperia A (2007) New roles for an old enzyme: Na,K-ATPase emerges as an interesting drug target. J Intern Med 261: 44–52. 17222167 10.1111/j.1365-2796.2006.01745.x

[21] Evans DH, Piermarini PM, Choe KP (2005) The multifunctional fish gill: dominant site of gas exchange, osmoregulation, acid-base regulation, and excretion of nitrogenous waste. Physiol Rev 85: 97–177. 15618479 10.1152/physrev.00050.2003

[22] Hwang PP, Lee TH (2007) New insights into fish ion regulation and mitochondrion-rich cells. Comp Biochem Physiol A Mol Integr Physiol 148: 479–497. 17689996 10.1016/j.cbpa.2007.06.416

[23] Lin YM, Chen CN, Lee TH (2003) The expression of gill Na, K-ATPase in milkfish, *Chanos chanos*, acclimated to seawater, brackish water and fresh water. Comp Biochem Physiol A Mol Integr Physiol 135: 489–497. 12829056 10.1016/s1095-6433(03)00136-3

[24] Crambert G, Geering K (2003) FXYD Proteins: new tissue-specific regulators of the ubiquitous Na,K-ATPase. Sci STKE 166: re1. 10.1126/stke.2003.166.re1 12538882

[25] Sweadner KJ, Rael E (2000) The FXYD gene family of small ion transport regulators or channels: cDNA sequence, protein signature sequence, and expression. Genomics 68: 41–56. 10950925 10.1006/geno.2000.6274

[26] Geering K (2006) FXYD proteins: new regulators of Na-K-ATPase. Am J Physiol Renal Physiol 290: F241–F250. 16403837 10.1152/ajprenal.00126.2005

[27] Garty H, Karlish SJD (2006) Role of FXYD proteins in ion transport. Annu Rev Physiol 68: 431–459. 16460279 10.1146/annurev.physiol.68.040104.131852

[28] Geering K (2005) Function of FXYD proteins, regulators of Na,K-ATPase. J Bioenerg Biomembranes 37: 387–392. 10.1007/s10863-005-9476-x 16691470

[29] Mahmmoud YA, Vorum H, Cornelius F (2000) Identification of a phospholemman-like protein from shark rectal glands. Evidence for indirect regulation of Na, K-ATPase by protein kinase C via a novel member of the FXYD family. J Biol Chem 275: 35969–35977. 10961995 10.1074/jbc.M005168200

[30] Mahmmoud YA, Cramb G, Maunsbach AB, Cutler CP, Meischke L, et al (2003) Regulation of Na,K-ATPase by PLMS, the phospholemman-like protein from shark: molecular cloning, sequence, expression, cellular distribution, and functional effects of PLMS. J Biol Chem 278: 37427–37438. 12874284 10.1074/jbc.M305126200

[31] Mahmmoud YA, Vorum H, Cornelius F (2005) Interaction of FXYD10 (PLMS) with Na, K-ATPase from shark rectal glands: close proximity of Cys^74^ of FXYD10 to Cys^254^ in the A domain of the α-subunit revealed by intermolecular thiol cross-linking. J Biol Chem 280: 27776–27782. 15919665 10.1074/jbc.M503150200

[32] Wang PJ, Lin CH, Hwang HH, Lee TH (2008) Branchial FXYD protein expression to salinity change and its interaction with Na^+^/K^+^-ATPase of the euryhaline teleost, *Tetraodon nigroviridis* . J Exp Biol 211: 3750–3758. 19011216 10.1242/jeb.018440

[33] Saito K, Nakamura N, Ito Y, Hoshijima K, Esaki M, et al (2010) Identification of zebrafish Fxyd11a protein that is highly expressed in ion-transporting epithelium of the gill and skin and its possible role in ion homeostasis. Front Physio 1: 129. 10.3389/fphys.2010.00129 PMC3059942 21423371

[34] Tipsmark CK (2008) Identification of FXYD protein genes in a teleost: tissue-specific expression and response to salinity change. Am J Physiol Regul Integr Comp Physiol 294: R1367–R1378. 18256141 10.1152/ajpregu.00454.2007

[35] Tipsmark CK, Mahmmoud YA, Borski RJ, Madsen SS (2010) FXYD-11 associates with Na^+^,K^+^-ATPase in the gill of Atlantic salmon: regulation and localization in relation to changed ion-regulatory status. Am J Physiol Regul Integr Comp Physiol 299: R1212–R1223. 20702795 10.1152/ajpregu.00015.2010

[36] Bystriansky JS, Schulte PM (2011) Changes in gill H^+^-ATPase and Na^+^/K^+^-ATPase expression and activity during freshwater acclimation of Atlantic salmon (*Salmo salar*). J Exp Biol 214: 2435–2442. 21697436 10.1242/jeb.050633

[37] Tipsmark CK, Breves JP, Seale AP, Lerner DT, Hirano T, et al (2011) Switching of Na^+^, K^+^-ATPase isoforms by salinity and prolactin in the gill of a cichlid fish. J Endocrinol 209: 237–244. 21330335 10.1530/JOE-10-0495

[38] Tang CH, Lai DY, Lee TH (2012) Effects of salinity acclimation on Na^+^/K^+^-ATPase responses and FXYD11 expression in the gills and kidneys of the Japanese eel (*Anguilla japonica*). Comp Biochem Physiol A Mol Integr Physiol 163: 302–310. 22885345 10.1016/j.cbpa.2012.07.017

[39] Takehana Y, Naruse K, Sakaizumi M (2005) Molecular phylogeny of the medaka fishes genus *Oryzias* (Beloniformes: Adrianichthyidae) based on nuclear and mitochondrial DNA sequences. Mol Phylogenet Evol 36: 417–428. 15955519 10.1016/j.ympev.2005.01.016

[40] Yang WK, Kang CK, Chen TY, Chang WB, Lee TH (2011) Salinity-dependent expression of the branchial Na^+^/K^+^/2Cl^−^ cotransporter and Na^+^/K^+^-ATPase in the sailfin molly correlates with hypoosmoregulatory endurance. J Comp Physiol B 181: 953–964. 21445564 10.1007/s00360-011-0568-0

[41] Hall TA (1999) BioEdit: a user-friendly biological sequence alignment editor and analysis program for Windows 95/98/NT. Nucl Acids Symp Ser 41: 95–98.

[42] Tamura K, Peterson D, Peterson N, Stecher G, Nei M, et al (2011) MEGA5: molecular evolutionary genetics analysis using maximum likelihood, evolutionary distance, and maximum parsimony methods. Mol Biol Evol 28: 2731–2739. 21546353 10.1093/molbev/msr121 PMC3203626

[43] Petersen TN, Brunak S, von Heijne G, Nielsen H (2011) SignalP 4.0: discriminating signal peptides from transmembrane regions. Nat Methods 8: 785–786. 21959131 10.1038/nmeth.1701

[44] Hirokawa T, Boon-Chieng S, Mitaku S (1998) SOSUI: classification and secondary structure prediction system for membrane proteins. Bioinformatics 14: 378–379. 9632836 10.1093/bioinformatics/14.4.378

[45] Blom N, Gammeltoft S, Brunak S (1999) Sequence- and structure-based prediction of eukaryotic protein phosphorylation sites. J Mol Biol 294: 1351–1362. 10600390 10.1006/jmbi.1999.3310

[46] Julenius K, Mølgaard A, Gupta R, Brunak S (2005) Prediction, conservation analysis and structural characterization of mammalian mucin-type O-glycosylation sites. Glycobiology 15: 153–164. 15385431 10.1093/glycob/cwh151

[47] Johansen MB, Kiemer L, Brunak S (2006) Analysis and prediction of mammalian protein glycation. Glycobiology 16: 844–853. 16762979 10.1093/glycob/cwl009

[48] Yang WK, Hseu JR, Tang CH, Chung MJ, Wu SM, et al (2009) Na^+^/K^+^-ATPase expression in gills of the euryhaline sailfin molly, *Poecilia latipinna*, is altered in response to salinity challenge. J Exp Mar Biol Ecol 375: 41–50.

[49] Feschenko MS, Donnet C, Wetzel RK, Asinovski NK, Jones LR, et al (2003) Phospholemman, a single-span membrane protein, is an accessory protein of Na,K-ATPase in cerebellum and choroid plexus. J Neurosci 23: 2161–2169. 12657675 10.1523/JNEUROSCI.23-06-02161.2003 PMC6742001

[50] Garty H, Lindzen M, Scanzano R, Aizman R, Füzesi M, et al (2002) A functional interaction between CHIF and Na-K-ATPase: implication for regulation by FXYD proteins. Am J Physiol Renal Physiol 283: F607–F615. 12217851 10.1152/ajprenal.00112.2002

[51] Béguin P, Crambert G, Monnet-Tschudi F, Uldry M, Horisberger JD, et al (2002) FXYD7 is a brain-specific regulator of Na,K-ATPase α1-β isozymes. EMBO J 21: 3264–3273. 12093728 10.1093/emboj/cdf330 PMC125393

[52] Li C, Grosdidier A, Crambert G, Horisberger JD, Michielin O, et al (2004) Structural and functional interaction sites between Na,K-ATPase and FXYD proteins. J Biol Chem 279: 38895–38902. 15234969 10.1074/jbc.M406697200

[53] Freire CA, Amado EM, Souza LR, Veiga MP, Vitule JR, et al (2008) Muscle water control in crustaceans and fishes as a function of habitat, osmoregulatory capacity, and degree of euryhalinity. Comp Biochem Physiol A Mol Integr Physiol 149: 435–446. 18325804 10.1016/j.cbpa.2008.02.003

[54] Cutler CP, Brezillon S, Bekir S, Sanders IL, Hazon N, et al (2000) Expression of a duplicate Na,K-ATPase β_1_-isoform in the European eel (*Anguilla anguilla*). Am J Physiol Regul Integr Comp Physiol 279: R222–R229. 10896885 10.1152/ajpregu.2000.279.1.R222

[55] Inoue K, Miyanishi H, Nobata S, Takei Y (2012) Evolutionary implication of the absence of atrial natriuretic peptide (ANP) in euryhaline *Oryzias* fishes. Environ Biol Fish 94: 559–566.

