# Supplementary material for: Expression Profiles of Branchial FXYD Proteins in the Brackish Medaka Oryzias dancena: A Potential Saltwater Fish Model for Studies of Osmoregulation
Source: PLoS One. 2013 Jan 31;8(1):e55470. doi: 10.1371/journal.pone.0055470 (PMC3561181; doi:10.1371/journal.pone.0055470)
Supplement: Table S1 — Accession numbers of FXYD proteins from the brackish medaka and Japanese medaka. (DOC) [file pone.0055470.s002.doc]

**Table S1. Accession numbers of FXYD proteins from the brackish medaka and Japanese medaka.**

| Proteins | Accession numbers | |
| --- | --- | --- |
|  | Brackish medaka | Japanese medaka |
| FXYD5 | JX569227 | ENSORLT00000008380 |
| FXYD6 | JX624723 | ENSORLG00000007198 |
| FXYD7 | JX624724 | JX565424 |
| FXYD8 | JX569228 | JX565423 |
| FXYD9 | JX569229 | JX565422 |
| FXYD11 | JX624725 | JX624726 |
| FXYD12 | JX569230 | JX643983 |

Accession numbers with the “JX” and “ENSORL” prefix are from the National Center for Biotechnology Information (<http://www.ncbi.nlm.nih.gov/guide/>) and the Ensembl (<http://www.ensembl.org/index.html>), respectively.
