# Supplementary material for: Expression Profiles of Branchial FXYD Proteins in the Brackish Medaka Oryzias dancena: A Potential Saltwater Fish Model for Studies of Osmoregulation
Source: PLoS One. 2013 Jan 31;8(1):e55470. doi: 10.1371/journal.pone.0055470 (PMC3561181; doi:10.1371/journal.pone.0055470)
Supplement: Table S2 — Predicted sequence information for FXYD proteins from the brackish medaka and Japanese medaka. (DOC) [file pone.0055470.s003.doc]

**Table S2. Predicted sequence information for FXYD proteins from the brackish medaka and Japanese medaka.**

| Proteins | ORF  (a.a / bp) | Signal peptide | Motif | TM (residue) | Phosphorylation site (residue) | Glycosylation site (residue) |
| --- | --- | --- | --- | --- | --- | --- |
| Brackish medaka (Od) | |  |  |  |  |  |
| OdFXYD5 | 176 / 531 | Yes | FKYD | 134-156 | Total: 3  S: 167, 168, 170 | Total: 31  T: 40, 42, 43, 49, 53, 54, 57, 63, 69, 73, 78, 83, 85, 93, 96, 101, 103, 107-110, 117  S: 51, 60, 68, 97, 102, 105  N: 36, 47, 67 |
| OdFXYD6 | 103 / 312 | Yes | FVYD | 44-65 | Total: 1  T: 99 | Total: 2  T: 99; N: 29 |
| OdFXYD7 | 72 / 219 | No | FDYD | 23-42 | Total: 4  S: 52, 53, 55, 57 | Total: 7  T: 3, 4, 6, 60, 65; S: 57; N: 58 |
| OdFXYD8 | 77 / 234 | Yes | FHYD | 38-59 | Total: 4  S: 65, 67, 70, 72 | Total: 0 |
| OdFXYD9 | 93 / 282 | Yes | FNFD | 41-62 | Total: 1  T: 76 | Total: 0 |
| OdFXYD11 | 71 / 216 | Yes | FVYN | 33-55 | Total: 3  S: 65, 67, 69 | Total: 0 |
| OdFXYD12 | 65 / 198 | No | FFYD | 26-48 | Total: 0 | Total: 0 |
|  |  |  |  |  |  |  |
| Japanese medaka (Ol) | |  |  |  |  |  |
| OlFXYD5 | 176 / 531 | Yes | FEYD | 135-157 | Total: 3  S: 167, 168, 170 | Total: 35  T: 40, 43, 48, 49, 54, 57, 63, 69, 72, 73, 78, 79, 83, 85, 93, 97, 99, 101, 103, 108, 109, 110, 117  S: 53, 60, 61, 68, 86, 88, 94, 104, 105,  N: 47, 67, 91 |
| OlFXYD6 | 100 / 303 | Yes | FIYD | 41-62 | Total: 1  T: 96 | Total: 1  T: 96 |
| OlFXYD7 | 72 / 319 | No | FEYD | 23-42 | Total: 4  S: 52, 53, 55, 57 | Total: 7  T: 3, 4, 60, 65; S: 55, 57; N: 58 |
| OlFXYD8 | 77 / 234 | Yes | FHYD | 38-59 | Total: 3  S: 65, 70, 72 | Total: 1  S: 72 |
| OlFXYD9 | 87 / 264 | Yes | FNFD | 35-56 | Total: 1  T: 70 | Total: 0 |
| OlFXYD11 | 70 / 213 | Yes | FVYN | 33-55 | Total: 2  S: 65, 68 | Total: 0 |
| OlFXYD12 | 66 / 201 | No | FFYD | 26-48 | Total: 1  Y: 66 | Total: 0 |

ORF, open reading frame; a.a., amino acid; bp, base pair; TM, transmembrane domain; T, threonine; S, serine; Y, tyrosine; N, asparagine.
