# Supplementary material for: Expression Profiles of Branchial FXYD Proteins in the Brackish Medaka Oryzias dancena: A Potential Saltwater Fish Model for Studies of Osmoregulation
Source: PLoS One. 2013 Jan 31;8(1):e55470. doi: 10.1371/journal.pone.0055470 (PMC3561181; doi:10.1371/journal.pone.0055470)
Supplement: Table S3 — FXYD protein accession numbers. (DOC) [file pone.0055470.s004.doc]

**Table S3. FXYD protein accession numbers.**

| Protiens | Accession numbers |  | Protiens | Accession numbers |
| --- | --- | --- | --- | --- |
| Teleosts |  |  |  |  |
| Zebrafish (*Danio rerio*, Dr) | |  | Atlantic salmon (*Salmo salar*, Ss) | |
| DrFXYD2 | XP_001334496.1 |  | SsFXYD2 | DAA06140.1 |
| DrFXYD5 | XP_706414.3 |  | SsFXYD5a | DAA06141.1 |
| DrFXYD6 | NP_956141.1 |  | SsFXYD6 | DAA06129.1 |
| DrFXYD7 | XP_001338521.1 |  | SsFXYD7a | DAA06133.1 |
| DrFXYD8 | AI958251.1 |  | SsFXYD8 | DAA06130.1 |
| DrFXYD9 | AW455046.1 |  | SsFXYD9a | DAA06131.1 |
| DrFXYD11 | XP_003200301.1 |  | SsFXYD11a | DAA06135.1 |
| DrFXYD12 | XP_002664620.1 |  | SsFXYD12a | DAA06137.1 |
|  |  |  |  |  |
| Nile tilapia (*Oreochromis niloticus*, On) | |  | Spotted green pufferfish (*Tetraodon nigroviridis*, Tn) | |
| OnFXYD6 | XP_003450861.1 |  | TnFXYD9 | ABL74277.1 |
| OnFXYD7 | XP_003454408.1 |  |  |  |
| OnFXYD8 | XP_003454406.1 |  | European eel (*Anguilla anguilla*, Aa) | |
| OnFXYD9 | XP_003443733.1 |  | AaFXYD11b | eu_c14786 |
| OnFXYD11 | XP_003443732.1 |  |  |  |
| OnFXYD12 | XP_003450860.1 |  | Japanese eel (*Anguilla japonica*, Aj) | |
|  |  |  | AjFXYD11 | AFK24487.1 |
| Elasmobranch |  |  |  |  |
| Spiny dogfish (*Squalus acanthias*, Sa) | |  |  |  |
| SaFXYF10 | P82542.2 |  |  |  |
|  |  |  |  |  |
| Mammals |  |  |  |  |
| Human (*Homo sapiens*, Hs) | |  | Mouse (*Mus musculus*, Mm) | |
| HsFXYD1 | NP_068702.1 |  | MmFXYD1 | EDL23964.1 |
| HsFXYD2 | NP_001671.2 |  | MmFXYD2 | EDL25650.1 |
| HsFXYD3 | NP_005962.1 |  | MmFXYD3 | EDL23956.1 |
| HsFXYD4 | NP_775183.1 |  | MmFXYD4 | EDK99588.1 |
| HsFXYD5 | NP_054883.3 |  | MmFXYD5 | EDL23967.1 |
| HsFXYD6 | NP_071286.1 |  | MmFXYD6 | AAH42579.1 |
| HsFXYD7 | NP_071289.1 |  | MmFXYD7 | EDL23965.1 |

The “eu” prefix indicates accession numbers from the EeelBase (<http://compgen.bio.unipd.it/eeelbase/>); other letters indicate accession numbers from the National Center for Biotechnology Information (<http://www.ncbi.nlm.nih.gov/guide/>).
