# Supplementary material for: Expression Profiles of Branchial FXYD Proteins in the Brackish Medaka Oryzias dancena: A Potential Saltwater Fish Model for Studies of Osmoregulation
Source: PLoS One. 2013 Jan 31;8(1):e55470. doi: 10.1371/journal.pone.0055470 (PMC3561181; doi:10.1371/journal.pone.0055470)
Supplement: Table S4 — Primer sequences and probe construction used for RACE, RT-PCR, and WISH of fxyd genes of the brackish medaka (Od) and Japanese medaka (Ol). (DOC) [file pone.0055470.s005.doc]

**Table S4.** Primer sequences and probe construction used for RACE, RT-PCR, and WISH of *fxyd* genes of the brackish medaka (Od) and Japanese medaka (Ol).

| Genes/ Applications | Fish | Primer sequence (5' to 3') | |
| --- | --- | --- | --- |
|  |  | Forward | Reverse |
| *fxyd5* |  |  |  |
| RACE | Od | GCGACCTGGAACACAAATGACTCAC | CAGCAGTTCGCAGGGACTCGTAATC |
| RT | Od, Ol | CCGGAACACAAATGACTCAC | ATCCTTGGACAACACGATATGA |
| *fxyd6* |  |  |  |
| RACE | Od | GAGAATTGGAGGACTGGCCTTTGC | CAGTGGCATCGCCGACTAAGAATAAG |
| RT | Od | TCACTCCTGGTATGCGTGTC | TCAGCTTCCGCTCTTAACCT |
|  | Ol | CAACCCTCCACTACATGAAACA | AAGCTAGTTGCAGTCCTGCTG |
| *fxyd7* |  |  |  |
| RACE | Od | CCACCACAGAAACCATGTTTCCAGAC | CACATTTTCTCCATGTGTGCATGAGTC |
| RT | Od | CACCACCAACTATGCACCAG | ACATGCTGCCAGTTCCTGTA |
|  | Ol | AACCTTGCGGACAACAGG | AAGGTTTCATAGTTACGATAGCACAA |
| *fxyd*8 |  |  |  |
| RACE | Od | GGATCTCATTGTGTTGGTGGCATTC | TGCTGCCATCTAGTGGTGACAATCAG |
|  | Ol |  | GCCATTACACTTCCGGATGACGAG |
| RT | Od | TCATTGTGTTGGTGGCATTC | TGGTGTCTTAAGAACTCCTTGTG |
|  | Ol |  | AATTGCACAGATGCTCATGC |
| *fxyd9* |  |  |  |
| RACE | Od | GCGCTTTGGTGCTGATGACACTC | TTCTGTCCGTTGCACGTTTGAGAAG |
| RT | Od, Ol | GAAGATCTGCGCTTTGGTG | TGGAGGTACAGGCTGCAGAT |
| *fxyd11* |  |  |  |
| RACE | Od | AGCGGACTCTGTGTTGGTGAAGATG | TTCATTTGCCACCATGTACCACAGC |
|  | Ol |  | CAGCTATCAACAGGCCGACAATGAC |
| RT | Od | GGCTCGTCATTGTCTGCTTG | GTCAGATCGCACTGCTAGAATC |
|  | Ol | AACCAGGTGGTGTCTTGGAG | GCATCAGATCCCACTGTCAT |
| WISH | Od | AACCAGACGGTGTCTTGGAG | TGTTCAGCTGGTTCGGTAATG |
| *fxyd12* |  |  |  |
| RACE | Od | ATAGATCCAGAATTGGACGCCGACT | GCTTGCCACATCTTCTGATCTTGTTG |
|  | Ol | GACGAGGCAGCATGTCGTCTTTG |  |
| RT | Od | TGCACACACTTCCAGACAGG | CCACATCTTCTGATCTTGTTGC |
|  | Ol | GCAGCTCAGCTATCTTCTCTGAA |  |
| *rpl7* |  |  |  |
| All PCR | Od, Ol | GTTCTGCAGCTTCTCCGTCT | GAGCTCTCGCACAGACTTCA |
