# Supplementary material for: Expression Profiles of Branchial FXYD Proteins in the Brackish Medaka Oryzias dancena: A Potential Saltwater Fish Model for Studies of Osmoregulation
Source: PLoS One. 2013 Jan 31;8(1):e55470. doi: 10.1371/journal.pone.0055470 (PMC3561181; doi:10.1371/journal.pone.0055470)
Supplement: Table S5 — Probe construction used for Q-PCR of fxyd genes of the brackish medaka and Japanese medaka. (DOC) [file pone.0055470.s006.doc]

**Table S5. Probe construction used for Q-PCR of *fxyd* genes of the brackish medaka and Japanese medaka.**

| Genes | Applications | Probe construction (5' to 3') | |
| --- | --- | --- | --- |
|  |  | Brackish medaka | Japanese medaka |
| *fxyd5* | QPCR-F | ACAGCCTGCGTGGATGAG | GTTGGATGAGGAGGAAGTGG |
|  | QPCR-R | TAAGCGTGGTGAGCAGGAAC | TTGTGTTCCAGGTCGCATC |
| *fxyd6* | QPCR-F | TCACTCCTGGTATGCGTGTC | GTGTGCGTGTCAGCTGTTG |
|  | QPCR-R | AGGCCAGTCCTCCAATTCTC | AGAATTGGAGGACTGGCCTT |
| *fxyd7* | QPCR-F | CTCCAAATCCAATTCCAACG | AACCTTGCGGACAACAGG |
|  | QPCR-R | CTGGTGCATAGTTGGTGGTG | CGCGACAAACAAAACAACTG |
| *fxyd*8 | QPCR-F | CTCCTGCACTCGCTTCAG | TCATTGTGTTGGTGGCATTC |
|  | QPCR-R | AGGAGGCAGAGTAGGACTGC | ACCGATTCGCAGAGATTCA |
| *fxyd9* | QPCR-F | TGCAAGTTCAACCAGGACAA | GAAGATCTGCGCTTTGGTG |
|  | QPCR-R | TTGGAGCTTAGCAGTTGCAG | ACCGCAGCAAGAATGAGG |
| *fxyd11* | QPCR-F | GGCTCGTCATTGTCTGCTTG | CGGACTCTGTGTTGGTGAAG |
|  | QPCR-R | GGTCAGATCGCACTGCTAGA | ATGACGAGGCCTCCAATTC |
| *fxyd12* | QPCR-F | GGCGTTGTTGTGTTCTTGTC | GACAGACGAGGCAGCATGT |
|  | QPCR-R | CTCAGTGCAGCTCAGTCATC | TCTGATCTTTGCTGGCATTG |

F, forward; R, reverse.
